# Neurotoxicity by mercury is not associated with autism spectrum disorders in Spanish children

**DOI:** 10.1186/s13052-020-0780-1

**Published:** 2020-02-12

**Authors:** Fernando Gil-Hernández, Antonio R. Gómez-Fernández, María Josede la Torre-Aguilar, Juan L. Pérez-Navero, Katherine Flores-Rojas, Pilar Martín-Borreguero, Mercedes Gil-Campos

**Affiliations:** 1 Department of Legal Medicine and Toxicology, School of Medicine, Granada University, Granada, Spain; 2 Department of Pediatrics, Reina Sofía University Hospital, University of Córdoba, Instituto Maimónides de Investigación Biomédica (IMIBIC), Córdoba, Spain; 3 Center for Biomedical Research on Rare Diseases (CIBERER-ISCIII), Madrid, Spain; 4 Pediatric Metabolism Unit, Reina Sofía University Hospital, Instituto Maimónides Investigación Biomédica(IMIBIC), Córdoba University, CIBEROBN, Madrid, Spain; 5 Unit of Psychology and Pediatric Psychiatry, Reina Sofía University Hospital, Córdoba, Spain

**Keywords:** Autism spectrum disorder, Children, Hair, Mercury, Neurotoxicity, Urine

## Abstract

**Background:**

The pathophysiological etiologies related with the development of Autism Spectrum Disorders (ASD) remain controversial. Different authors have studied neurotoxins such as mercury (Hg) and their relationship with ADS. The objective of this study was to assess the levels of Hg in hair in a group of ASD children (chronic exposure) and in urinary excretion (acute exposure), in comparison to a healthy group.

**Methods:**

A case-control study was conducted in Spanish children. We compared 54 ASD children (aged 2–6) with no other associated pathology to a normally-developing control group (54 subjects).

**Results:**

There were no differences in urine (p:0.631) and hair (p:1.000) samples percentages below the limits of detection between the control and the ASD groups, and also between patients in the regression ASD subgroup (AMR) (p:0.08) and the non-regression ASD subgroup (ANMR) (p:0.705). When the analysis was adjusted for age and sex, the differences between Hg levels maintained not significant. There were no correlations between Hg concentrations in the ASD group as a whole (p: 0.739), or when they were subdivided into ASD-AMR (p: 0.739) and ASD-ANMR (p: 0.363).

**Conclusions:**

The present study shows no evidence in our geographical area to support an association between mercury neurotoxicity and the etiopathogenesis of ASD.

## Background

Environmental factors have been involved in the pathogenesis of Autism Spectrum Disorders (ASD). However, the role of heavy metals has not been fully defined. One of the etiopathogenic hypotheses put forward to account for ASD suggests the possibility of neurotoxicity deriving from heavy metals, especially mercury (Hg). Hg is a unique element that, unlike many metals, has no essential biological function in humans. Its presence in the body is exogenous and mainly comes from the intake of contaminated food, especially fish caught or farmed industrially. Hg content in hair is particularly related to dietary exposure mainly fish consumption [1]. Non-oxidized mercury is highly liposoluble with the capacity to cross the blood-brain barrier and cause neuronal toxicity.

Children are more susceptible than adults to environmental contaminants. They possess immature detoxification mechanisms and their heightened vulnerability is related to greater surface area relative to mass, nutritional aspects (children drink and eat more per unit of body weight) and behavioral patterns (mouth-touching behaviors) [2, 3]. All these hypotheses suggest the possibility of defects in the processes of metabolizing and eliminating heavy metals in children with ASD, contributing to the development of symptoms.

Although blood and urine analyses have been the traditional methods used for biomonitoring, human hair provides an interesting matrix as concentrations of metal compounds are up to 10 times higher than when they are found in blood or urine samples. Complementary to blood and urine concentrations, which display recent exposure, hair levels reveal an accumulated past exposure, providing an average of the growth period [4]. Therefore, hair testing can be utilized as a screening tool for biomonitoring chronic exposure to toxic substances in the environment.

Some of the published studies have significant limitations with controversial results, including lack of replication, limited sample sizes, retrospective design, recall and publication biases, inadequate matching of cases and controls, and the use of nonstandard tools to diagnose ASD [5]. A recent systematic reviewhas offered evidence that exposure to environmental toxins throughout the different developmental stages may increase the risk for developing ASD, and that environmental and genetic risk factors contribute to the development of ASD [5]. Results from a meta-analysis revealed that mercury could be an important causal factor in the etiology of ASD. It appears that the detoxification and excretory mechanisms are impaired in ASD patients, leading to the accumulation of mercury in the body [6]. Statistically significant correlations between increased Hg concentrations in hair and increased ASD severity have been also revealed [7]. Concentrations of blood and of urine along with hair could serve in the prediction of possible health effects as outcomes of the different forms of exposure to Hg as in ASD [8–11]. Thus the aim of the present study was to assess mercury levels in hair and urinary excretion levels of children with primary or idiopathic ASD and in healthy children of similar age. All of the children in the study were collected from the same geographical area and had similar demographic, socioeconomic and housing characteristics, as well as environmental and domestic exposure. The diagnosis of ASD was made by highly experienced professionals during the first years of life, since this is a very sensitive stage of neurodevelopment.

## Materials and methods

### Subjects

An observational case-control study with pre-scholar children (aged 2–6 years old) with an ASD diagnosis according to the DSM-5. Children were consecutively selected and excluded children with other pathologies non-ASD related, within a different age range or not complying with a similar environment. In addition, children were not considered for the study if they had been given mercury dental amalgams or had been treated with chelators.

Children with ASD were selected to assess Hg concentrations in hair and urine samples, and to investigate a possible relationship with ASD core symptoms and other developmental parameters.Hg levels in children with ASD were compared to Hg levels in a control group of normally-developing children living in the same urban area of Córdoba (Spain), with families in a similar economic and social situation with similar environmental exposure that the ASD group.

All the cases of ASD were selected after carrying out the Checklist for Autism in Toddlers (M-CHAT). Afterwards, a thorough clinical history of each child was obtained. The Autism Diagnostic Observation Schedule, Second Edition (ADOS-2), was used in suspected cases of ASD. At the same time, each of these subjects performed the following tests: the Autism Diagnostic Interview-revised (ADI-R) [12]. The Pervasive Developmental Disorders Behavior Inventory™ (PDDBI) [13], the Childhood Autism Rating Scale test (CARS), the Battelle Developmental test, and the Strengths and Difficulties Questionnaire (SDQ). These tests allowed us to diagnose ASD with a greater degree of certainty.

All the ASD cases selected in our study did not present any other associated pathology (seizures, or other neurological, metabolic or genetic diseases). Each patient was clinically assessed with complementary explorations. As well, children underwent a genetic study (karyotype and microarrays) to detect secondary or syndromic ASD. None of the patients required medication to treat behavioral disorders or aggressiveness.

Given that up to a third of patients with autism spectrum disorder (ASD) present neurodevelopmental regression and exhibit loss of previously acquired language and social skills, the group of ASD children was further subdivided into two subgroups, based on presence (AMR) or absence of neurodevelopmental regression (ANMR) during the first years of life.

An initial developmental clinical interview (adapted from a number of sources including the ADI-R and the Parent Interview for Autism – Clinical Version) was undertaken to identify the core symptoms of ASD and neurodevelopmental regression [14].

The control group consisted of children with the same demographic, environmental exposure and household characteristics as the ASD group. They also had the same immunizations, according to the vaccination schedule in our region. The clinical and analytical absence of illness in these children was confirmed. Informed written consent of the children’s legal guardians was obtained. This study was approved by the Hospital Biomedical Ethics Committee and conformed to the ethical standards laid down in the 1964 Declaration of Helsinki.

### Clinical history

Several questionnaires were administered to the parents or legal guardians of the children selected to participate in order to gather information about the demographic, socioeconomic and housing characteristics, as well as the environmental and home exposures, the parents’ occupational history, maternal dental work, and whether Rho (D) immune globulin was given during pregnancy, among other variables. A detailed developmental clinical medical history, including major childhood illnesses and immunizations, potential environmental toxic exposures and dietary anamnesis was obtained from all the children. Also a standard validated questionnaire was made to obtain information about frequency of food intake and food habits in both study groups. Physical exhaustive examination was also carried out, focusing on the child’s neurological and nutritional status. Anthropometric measurements (weight, height, body mass index [BMI]) were obtained using standard techniques.

### Urine sample

A first morning urine sample was collected from each participant in a sterile polyethylene container pre-treated with HNO3 (20%) and rinsed twice with MilliQ water. Samples were then stored at − 80 °C until analyzed. Major biomonitoring studies conducted in children (e.g., NHANES, GerES studies) have used urine instead of invasive samples, such as blood, as the latter are difficult to obtain and would result in low participation rates potentially leading to selection bias [15]. Given that the World Health Organization guidelines consider that urine samples with creatinine values less than 0.3 g/l or greater than 3 g/l are unrepresentative, they should be excluded from statistical analysis to minimize both over- and underestimation in calculating normalized concentrations.

### Hair sample

Human hair was cut from the occipital region. A minimum of 30 mg of hair was required for the analysis. Hair was submitted to ultrasonic cleaning in a non-ionic detergent and subsequently with an ethanol solution.

### Determination of mercury

Validation of analytical procedures used for the determination of metal compounds in urine and hair samples has been reported elsewhere [16]. The analytical method was controlled by using external certified reference materials (CRM). The reference sample for urine (ref. 201,205) was supplied by Seronorm (Billingstad, Norway), and the reference material NIES No. 5 for hair was obtained from the National Institute for Environmental Studies, Japan Environment Agency. In addition, the authors enlisted the help of an external Quality Control for Hg in urine provided by the Instituto Nacional de Seguridad e Higiene en el Trabajo, Ministry of Labor, Spain. The Atomic Absorption Spectrometry-furnace technique was employed for Hg determination using a hydride generation system. Urinary creatinine levels were determined by the Jaffe method using a Hitachi 917 auto-analyzer. Limits of detections (LOD) for urine (μg L-1) and hair (μg g-1) were 0.002 and 0.0002. Limits of quantifications (LOQ) for urine (μg L-1) and hair (μg g-1) were 0.007 and 0.0007. The coefficient of variation in the analysis was 3.56% [16].

### Statistical analysis

Hg levels in urine were used to estimate sample size considering the ASD group (AMR; ANMR) and the control group. Accepting an alpha risk of 0.05 and a beta risk of 0.2 in a two-sided test, 19 subjects in each group were necessary to recognize a minimum difference of 3 μg-L in Hg levels as statistically significant. Common standard deviation was assumed to be 1.5. A drop-out rate of 20% was anticipated. Geometric means and percentiles were calculated to describe metal concentrations in samples. Levels below the Limits of detection (LOD) were assigned the LOD (expressed as μg L-1 creatinine and μg g-1 for urine and hair samples, respectively), and divided by the square root of 2.

Differences in mean levels of mercury, as well as the potential influence of predictor variables and potential confounders (sex, age, BMI) were assessed by the Mann-Whitney’s test and the Kruskal–Wallis tests (for non-normal distribution), or with the Student’s test (for normal distribution). Categorical data were analyzed using the Chi-square test. The magnitude of the correlation between metal concentrations in urine and hair samples was assessed by the Spearman rank correlation test. Univariate and multivariate Tobit log-linear regression models were used to evaluate variables associated to metal levels. The use of Tobit models allows for an estimate of the effect, despite having a large number of samples below the LOD.

As recommended by Barr et al. [17] urine creatinine levels were included as an independent covariate in the regression models, rather than using creatinine-adjusted metal concentrations (creatinine standardization), because this approach is less prone to producing biased effect estimates. The statistical analyses were performed using the SPSS 21 software and the State 11 statistical package software.

## Results

Fifty-four healthy children as controls and 57 patients with ASD were assessed. Three patients in the ASD group did not meet the clinical criteria for ASD during the subsequent interviews. Therefore, they were excluded from the study. All the ASD children in the study exceeded the cut-off points for the diagnosis of ASD on the ADOS-2 test. Neither they nor their mothers had been subjected to any known toxic exposure.Two ASD boy could not be classified because he had come from national adoption at 20 months. All the children included in the study had received routine childhood vaccinations and neither they nor their mothers had been subjected to any known toxic exposure.

The general characteristics of the ASD children and those of the normally-developing controls are shown in Table 1. Children in the control and the ADS groups were selected within a similar age range. We did not find any significant differences between the groups regarding weight, height and body mass index [BMI]). The ASD group comprises a higher percentage of boys, as expected. As well, we did not find any differences between the groups regarding BMI.After administering the regressive classification test to the ASD group, it was determined that 19 patients met the AMR criteria and 33 met the ANMR criteria. In relation to fish intake by week, no differences were found between the control and the ASD groups (Table 1), including the AMR and ANMR subgroups (p: 0.273).

**Table 1:** Demographic data and fish intake frequency from children with autism spectrum disorders (ASD) compared with a group of healthy children (control group)

	ASD N: 54	Control N: 54	*p*
Sex (male)	45 (83%)	30 (56%)	0,002
Body mass index (kg/m^2^)	15,9 ± 1,7	18,1 ± 1,7	0,509
Fish intake (per week) %			
0	7 (13%)	10 (18%)	0,109
1	31 (57%)	38 (71%)	
2	13 (24%)	6 (11%)	
3	3 (6%)	0	

Table 2 shows the percentiles and geometric means of Hg levels in hair and urine in both groups, as well as in the AMR and ANMR subgroups. There were no differences concerning the percentages below the LOD in urine (p: 0.631) and hair (p: 1) samples between controls and the ASD group, and also between children in the AMR and ANMR groups, respectively (p: 0.08; p: 0.705). Furthermore, there were no differences in Hg levels in urine and hair by sex in the ASD group (urine: male: 0.50 ± 0.62 μg/L vs female: 0.76 ± 1.31μg/L; p: 0.990; hair: male: 7.8 ± 9.38 μg/g vs female: 11.23 ± 15.19 μg/g; p: 0.685), as in the control group (urine: male: 0.252 ± 0.34 μg/L vs female: 0.43 ± 0.49μg/L; p: 0.313; hair: male: 11.33 ± 12.19 μg/g vs female: 15.08 ± 13.23 μg/g; p: 0.990). When the analysis was adjusted for age and sex in the multiple log-linear regression study, the Hg differences in urinary levels and hair concentrations were not significant (Table 3).

**Table 2:** Concentrations for mercury (Hg) in urine and hair samples from children with autism spectrum disorders (ASD) and a healthy children control

Heavy metal	Groups	% LOD	AM ± SD	GM	Median (IQR)	Maximum
Hg Urine (ug/L)	Control (*n* = 54)	25(46%)	0,33 ± 0,42	0,04	0,17 (0,002-0,58)	1,39
	ASD	26 (51%)	0,54 ± 0,78	0,03	0,002 (0,002-0,98)	3,86
	ANMR(*n* = 33)	12 (39%)	0,68 ± 0,85	0,08	0,55 (0,002-1,18)	3,86
	AMR(*n* = 19)	13 (68%)	0,35 ± 0,62	0,01	0,002 (0,002-0,55)	1,90
Hg Hair (ug/g)	Control (*n* = 54)	2 (4%)	13 ± 12,68	5,40	10,08 (3,13-18,2)	51,99
	ASD	2 (4%)	8,26 ± 10,57	3,21	4,46 (1,66-9,35)	48,94
	ANMR (*n* = 33)	2 (6%)	9,26 ± 12,34	2,66	4,46 (1,66-9,78)	48,94
	AMR (*n* = 19)	0	6,57 ± 6,81	4,13	3,79 (1,63-9,33)	27,03

LODs in urine ug /L and hair (ug/g) were 0.002 and 0.0002 for Hg

*LOD* Limits of detection, *AM* mean, *SD* standard deviation, *GM* geometric mean, *IRQ* interquartile range, *ASD* Autism spectrum disorders

**Table 3:** Multiple log-linear Tobit regression analysis of Hg levels in hair and urine

Biological sample	Predictor variable	Pseudo R^2^	β	*p*-value	CI 95%
Hair	Groups (ASD)	0,03	−5.02	0,19	−12.67; 2.63
	Gender (girls)		3.96	0,12	−1.11; 9,1
	Age (per month increase)		−0,03	0,62	−0,16; 0,09
Urine	Groups (ASD)	0,05	−0.17	0,62	−4,97; 1,87
	Gender (girls)		0,24	0,31	−2,56; 1,04
	Age (per month increase)		−0,01	0,11	−0,002; 0,07

*ASD* Autism spectrum disorders, *CI* confidence interval

There were no correlations between Hg in hair and urine concentrations in the ASD group as a whole (p: 0.739; R: 0.033), or when they were subdivided into AMR (p: 0.739; R: 0.033) and ANMR (p: 0.363; R: 0.169). Multiple correlations were made between Hg levels and the Battelle, ADOS-2, PDDB, CARS, SDQ tests, including each of the areas that all these tests assess. There were also no correlations between Hg in hair and urine concentrations in the ASD group and the studied behavioral clinical scores.

All the children, regardless of which group they belonged to, scored above the cut-off point for the diagnosis of ASD. No differences were observed between the two subgroups of ASD in terms of the scores for the various areas assessed by the ADOS-2 test (results are not shown). On the other baseline tests, children with ASD in the AMR subgroup had significantly higher scores on the CARS test (ANMR: 30.6 ± 6.11 vs AMR: 35.9 ± 8.12; *p* = 0.009) and autism score on the PDBBI test (ANMR: 46.13 ± 10.47 vs AMR: 53.39 ± 10.39; *p* = 0.023). On the overall score of the Battelle Inventory, the AMR subgroup had lower score, which suggests less development (ANMR: 60.96 ± 13.25 vs AMR: 47.05 ± 10.33; *p* = 0.002).

## Discussion

In the current study, Hg concentrations in ASD children’s hair and urine were no different to the concentrations found in the group of normally-developing children. More than half of urine Hg determinations in the ASD and control groups were below the detection capacity. In hair, Hg concentrations in both groups were even much lower than the percentage of LOD. Moreover, we found no correlations between Hg concentrations in hair and urine in the ASD group and in the AMR and ANMR subgroups. So, these results indicate that Hg concentrations are very low in the hair of children with ASD, which expresses a very low chronic exposure, and also in urine concentrations, indicating very little current exposure to Hg. The worse scores obtained by the AMR group compared to the ANMR group on the CARS, SDQ and PDDBI tests may be due to a greater severity of the AMR group symptoms, without being attributed to mercury levels between both groups.

Some of the published studies have important limitations in the methodology with controversial results. Therefore, in this study, Hg in hair and urine was measured in a control group and in another of children with ASD from our same geographic area, with very similar demographic, socioeconomic and housing characteristics, as well as the same environmental exposure and domestic. The parents’ occupational history was similar in the children studied. Immunizations were the same. There were no environmental toxic exposures to heavy metals. Frequency of fish intake (Mediterranean eating pattern) and food habits were very similar in both the ASD and the normally-developing group of children. The diagnosis of ASD was made by highly experienced professionals during the first years of life of children, since this is a very sensitive stage of development.Regarding sex, in our prospective observational case-control study ASD prevalence varied by sex, being more frequent in men (Table 1). Our results are consistent with other published studies, in which males were until four times more likely than females to be diagnosed with ASD [18–21].

There were no environmental toxic exposures to heavy metals. Frequency of fish intake (Mediterranean eating pattern) and food habits were very similarin both the ASD and the normally-developing group of children.

By using a precise and validated methodology, we found no evidence between high concentrations of Hg and ASD symptomatology. On the contrary, Blaurock-Busch E et al. [11] have examined possible environmental risk factors and sources of exposure to Hg among other heavy metals in children with ASD vs controls. These authors have found a statistically significant difference in the mean urine levels of Hg, not in hair. A case-control study by Tabatadze T et al. [10] has assessed concentrations of heavy metals in samples of ASD children, and has carried out the identification of changes associated with ASD in 60 children aged from 4 to 5 years old. These authors obtained high association and significant values between Hg concentrations and ASD. These results also showed high contamination by heavy metals in ASD children, compared to healthy ones.

On the other hand, Qin YY et al. [8] have compared blood plasma in ASD children to that in their healthy counterparts, and have shown that ASD children exhibited higher Hg levels than healthy children. Positive associations were found between levels of Hg and seafood. Similarly, Li H et al. [9] have investigated whether blood levels of Hg and other heavy metals in ASD children significantly differ from those of age-and sex-matched controls. The results of these studies could support a key role for heavy metal exposure, namely Hg, in the genesis of ASD symptoms. However, it should be considered that the studies undertaken by said authors comprised cohorts that had been collected from a geographical and demographic area different to that of the children included in this study, where environment and eating habits greatly differ from those of the series studied by these authors.

Fang T et al. [22] examined the relationship between dietary Hg exposure and hair total mercury (THg) in an eastern China community where freshwater fish, as opposed to marine fish, dominates cuisine. Hair THg concentration was positively associated with the average mass of fish consumed weekly, indicating that fish consumption was the main contributor to hair THg in this geographic area, which influenced the differences in descriptive results. Moreover, there are multiple hypotheses to explain this pathology. In fact, our research group has recently reported significant differences in other biological parameters and has also observed intestinal microbiota changes between ASD patients [22–24].

Hg is oxidized in the erythrocytes following the action of glutathione (GSH), and is essentially eliminated by feces through bile, urine and by its incorporation into hair. One of the most-studied etiopathogenic hypotheses put forward to account for ASD is the possibility of heavy metal intoxication, especially Hg. Bernard et al. [25] elaborated the hypothesis that ASD was related to Hg toxicity, based on the similarity between chronic Hg poisoning symptoms and ASD core deficits. On their part, Nelson et al. [26] examined both variables and concluded that some of the manifestations of Hg intoxication, such as ataxia, visual disturbances, peripheral neuropathy, hypertension, rashes and thrombocytopenia are not exhibited in ASD. In this regard, there have been numerous child-based studies that have sought to clarify the degree of involvement of heavy metals in the development of ASD, with conflicting results stemming from the different populations used, and the associated factors analyzed in relation to the severity of the symptoms [27–29]. Many of the studies have significant methodological limitations, and in ASD children certain genetic and toxic environmental factors may have a synergic influence at critical stages of neurodevelopment. Although there are positive associations between ASD and heavy metal exposures in the environment, these authors cannot draw firm conclusions on causation [9].

It should also be pointed out that the safe limit of Hg is 1 μg/g [30] and the mean level for European children is 1.99 μg/g [31]. Some authors have shown positive associations between ASD and Hg toxicity (Hg levels measured only in hair) [22, 32] related to maternal fish consumption during pregnancy and the average mass of weekly fish consumed by autistic children. These studies have been carried out on Asian and African populations, and it appears that oceans and/or rivers involved are more contaminated, or that subjects consume larger amounts of fish. Detailed data on dietary intake primarily focusing on the types of fish and on quantification with validated questionnaires, have not been provided. Frequency of food intake was analyzed in our sample, especially in relation to fish consumption, and we observed no differences between healthy children and the group of children with ASD, with or without neurodevelopmental regression (Table 1). Consequently, it may be that there is a range of environmental exposures impinging on these results. In the present study, there are no significant differences regarding Hg concentrations in urine or hair in ASD children, compared to healthy children from the same geographical and environmental area.

The limitations of our study include the difficulty to collect a homogeneous sample of ASD and healthy children from the same geographical area and sharing similar socio-economic and environmental conditions. The selection bias in the diagnosis for ASD was avoided by using five specific tests, which were performed by qualified professionals to confirm ASD. The estimation of the sample was accordance with the sample size, with sufficient power to detect differences and associations. Moreover, we conducted a regression analysis adjusted for age and sex to avoid these possible confusing factors.

## Conclusions

In this observational case-control study, using a homogenous sample of Spanish children diagnosed with ASD compared with healthy similar children, there was no evidence to support a relationship between Hg concentrations in hair and urine and ASD in our geographical area. Despite these results, further high-quality epidemiological studies on environmental toxicants and ASD are warranted to clarify controversies in the published findings.

## Data Availability

The datasets used are available from PNJL on reasonable request.
